# KECA Similarity-Based Monitoring and Diagnosis of Faults in Multi-Phase Batch Processes †

**DOI:** 10.3390/e21020121

**Published:** 2019-01-28

**Authors:** Yongsheng Qi, Xuebin Meng, Chenxi Lu, Xuejin Gao, Lin Wang

**Affiliations:** 1 Institute of Electric Power, Inner Mongolia University of Technology, Hohhot 010080, China; 2 College of Electronic and Control Engineering, Beijing University of Technology, Beijing 100124, China

**Keywords:** KECA, fault monitoring, fault diagnosis, batch process, multi-stage

## Abstract

Multiple phases with phase to phase transitions are important characteristics of many batch processes. The linear characteristics between phases are taken into consideration in the traditional algorithms while nonlinearities are neglected, which can lead to inaccuracy and inefficiency in monitoring. The focus of this paper is nonlinear multi-phase batch processes. A similarity metric is defined based on kernel entropy component analysis (KECA). A KECA similarity-based method is proposed for phase division and fault monitoring. First, nonlinear characteristics can be extracted in feature space via performing KECA on each preprocessed time-slice data matrix. Then phase division is achieved with the similarity variation of the extracted feature information. Then, a series of KECA models and slide-KECA models are established for steady and transitions phases respectively, which can reflect the diversity of transitional characteristics objectively and preferably deal with the stage-transition monitoring problem in multistage batch processes. Next, in order to overcome the problem that the traditional contribution plot cannot be applied to the kernel mapping space, a nonlinear contribution plot diagnosis algorithm is proposed, which is easier, more intuitive and implementable compared with the traditional one. Finally, simulations are performed on penicillin fermentation and industrial application. Specifically, the proposed method detects the abnormal agitation power and the abnormal substrate supply at 47 h and 86 h, respectively. Compared with traditional methods, it has better real-time performance and higher efficiency. Results demonstrate the ability of the proposed method to detect faults accurately and effectively in practice.

## 1. Introduction

With the considerable advances of modern society and rapid market changes, there is a growing demand for a wide variety of high-quality products. Batch processes, characterized by flexibility, have been widely used in small-scale manufacturing of high-added-value products, and has become the major mode of production in the medical, biological and chemical fields. Therefore, developing an effective monitoring system for such processes is critical for the security and reliability of batch processes.

Most batch processes consist of multiple phases, each of which possesses its own characteristics and dominant variables [1]. Readers should note that the multiple phases mentioned in this paper are based on statistics and do not necessarily correspond to physical phases of the real process. For example, the data in the same phase have the same distribution or the variables have the same level of correlation. Currently, the traditional multi-way principal component analysis (MPCA) method has been successfully applied to monitoring batch processes [2,3,4]. If the monitoring model is established by considering a complete set of batch data as a statistical sample, local features of the behavior conducted during the batch process are ignored, making it hard to determine the change in correlation between procedure parameters. MPCA is a linear modelling method in nature and is thus ineffective for nonlinear multi-phase batch processes. Kosanovich proposed a two-phase MPCA algorithm [5], and pioneered the research of batch process phases. Lu et al. proposed a K-means clustering-based algorithm to automatically partition a batch process into several stable sub-phases [6], in which the time data was partitioned into corresponding sub-phases to produce desirable results. If several sub-phases are executed during the production process, it is impossible to make a direct transition from one steadily operating sub-phase to another. Therefore, it is insufficient to only focus on the stable sub-phases, since transitions between different stable sub-phases need attention as well. In [7], Zhao et al. proposed an improved multiphase method which considers both hard partitioning and soft transition between different phases. An automatic step-wise sequential phase partition (SSPP) algorithm was also developed to overcome the problem of hard-partition and disorder in clustering-based division algorithm [8]. Hu et al. proposed a novel multi-phase monitoring method, based on batch weighting, soft classification, auto-regression and principal component analysis [9]. It alleviates false and missing alarms of the traditional method, where only hard dividing of the process is considered and the process is modelled without taking the dynamics of the process into account.

All the methods mentioned above study the variation of process correlation within the execution time, with the help of which infer the variation of the process’ inherent operation mechanism. Specifically, the process features are extracted from time-slice matrices of batch processes through PCA, forming the so-called loading matrices which are fed as the input of the clustering algorithm. Alternatively, the sub-phases are partitioned or modelled using the correlation among the neighboring time slice load matrices. However, the PCA method can only extract the linear features of processes, without considering nonlinearities between variables. Therefore, this kind of method is inappropriate for complex batch processes with heavy nonlinearities. In contrast, the kernel entropy component analysis (KECA) method is able to extract non-linear features effectively via the non-linear mapping method. Note that the non-linear function is unknown during the kernel mapping. Therefore, most of the clustering algorithms require the information about the loading matrices, particularly when calculating the cluster center. As a result, unlike the linear methods, during the partitioning of the nonlinear batch process into sub-phases, the loading matrices cannot be fed directly as the input of the clustering algorithm. To tackle this problem, in this context, a KECA-based similarity metric is defined and a KECA similarity-based nonlinear clustering algorithm is proposed.

Among the various models available for monitoring the faults of batch processes, the PCA method has been widely used in practical applications [10,11]. However, the PCA algorithm is based on the assumption that the production process is linear. However, this assumption is invalid for the complex and non-linear batch processes which reduces its performance to a great extent. In [12], Scholkopf et al. proposed kernel principal component analysis (KPCA). In this method, the original input region is first mapped to the high-dimensional feature space through kernel mapping. PCA is then performed in this high-dimensional feature space. In this way, the non-linear data from the input region is transformed into the linear region in the feature space [13], providing a viable approach to solving data non-linearity. Based on KPCA, Jenssen proposed the KECA algorithm for data conversion and data dimensionality reduction [14], which showed the unique superiority in terms of data feature extraction. Compared with the traditional KPCA, the criterion for the choice of the principal component in KECA is the amount of information entropy rather than the contribution of the variance of feature data. The influence of the eigenvalues and the feature vectors on the monitored results is taken into account. It is proven that the principal component selected by KECA have a rigid angular structure, i.e., the principal components of different types of data concentrated in the neighborhood of different coordinate axes, thereat facilitating data classification [15]. In this paper, the KECA method is used to choose the principal component’s angular structure represented by a divergence statistic. With this statistic in hand, the similarity under probability density distributions can be represented more effectively to distinguish from anomalous distribution [16].

The commonly used methods for fault detection include the contribution plotting and the fault reconstruction. Li et al. [17] did a review of recent progress on decoupling diagnosis of hybrid failures in gear transmission systems using vibration sensor signal and a potential methodology based on the bounded component analysis (BCA) for hybrid faults decoupling was discussed. In [18], Rostami et al. firstly applied the well-known support vector machine (SVM) classifier to detect the abnormal observations. Fault fingerprints can be extracted by principal component analysis (PCA). This research focused on proposing an efficient data-driven fault diagnostic method to monitor the equipment condition, and consequently to detect and classify the faults. In [19], Nguyen et al. proposed a data-driven prognostic method for BMP organized in three steps. The emphasis is on the use of the percentile measure to process the raw health index. The remaining useful life (RUL) is then estimated using an aggregate probability density function (pdf) with a confidence interval (CI). The proposed method is applied on semiconductor manufacturing equipment. In [20], Wang G. introduced the contribution plot for fault diagnosis. However, in the traditional methodology, the plot represents the contribution of the original measuring variables to the monitoring statistic. It is thus necessary to derive corresponding formula of the contribution for each given monitoring method. For complicated fault monitoring methods (e.g., kernel entropy learning), it is very hard or even impossible to construct an appropriate formula to compute the contribution, which enormously limits the application of the contribution plot [21]. Yue et al. [22] proposed a fault diagnosis method based on fault reconstruction. Unlike the contribution plot, it requires a large amount of historical data for various faults, before identifying the procedure parameter of the fault. In order to address the limitations of the contribution plot and fault reconstruction method, a standard vector kernel contribution plot (SV-KCP) method is proposed in this paper. The monitoring samples collected at the moment of fault are directly reconstructed. After the diagnosis is determined at a specific moment, all variables of the fault samples are used to substitute the corresponding standard samples. Their statistics are computed accordingly to determine the procedure variables that could cause the fault. The proposed method provides a quantitative description via the histogram, which makes it easier, more intuitive and practical to use.

To sum up, this paper focuses on a complete multiphase strategy for monitoring and diagnosing the fault of complicated batch processes with heavy nonlinearities. The phases are partitioned by using a KECA-based similarity metric, and then KECA models and slide-KECA models are established for steady phases and transitions respectively. After that, a SV-KCP-based fault diagnosis method is proposed to locate the fault causing variables via the kernel plotting. Experiments are performed on the penicillin ferment to highlight the advantages of the proposed method.

**Figure 1:**
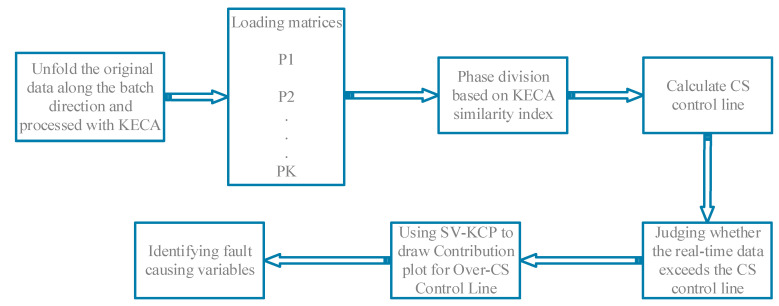
The overall block diagram of the algorithm.

This article is organized as follows: a KECA based similarity metric is defined in Section 2 and simultaneously a phase partitioning algorithm is proposed. In Section 3, corresponding monitoring models are established for each steady phases and transitions, and CS statistic is introduced for fault diagnosis. SV-KCP method is also presented in Section 3. Simulation results and industrial application results are exhibited to validate the effectiveness of the proposed method in Section 4 and Section 5, respectively. Ultimately, conclusions are drawn in Section 6. An overall block diagram of the algorithm in this paper is shown in Figure 1.

## 2. Phase Partitioning Based on KECA Similarity

### 2.1. KECA

Kernel entropy component was first proposed by Jenssen [14] in 2010. It was underpinned by two concepts: Renyi entropy and Parzen window density estimation. The first one can be written as:

(1) $$\hat{V}\left(x\right)=-\log V\left(p\right)=-\log\int p^{2}\left(x\right)dx,$$

and the second can be written as:

(2) $$\hat{p}\left(x\right)=\frac{1}{N}\sum_{x_{i}\in D}k_{\sigma }\left(x,x_{i}\right),$$

where *x* denotes the sample, *V*(*p*) = *ε_p_*(*p*), *ε_p_*(•) denotes expectation with regard to the density *p*(*x*), *N* denotes its dimensionality, *p*(*x*) is the probability density function of *x*, $k_{\sigma }\left(x,x_{i}\right)=\exp\left(-\frac{\left\|x-x_{i}\right\|}{2\sigma^{2}}\right)$, whose width is controlled by the parameter σ. By approximating $\hat{V}\left(p\right)$ with the mean value, we have:

(3) $$\hat{V}\left(p\right)=\frac{1}{N}\sum_{x_{t}\in D}\hat{p}\left(x_{i}\right)=\frac{1}{N^{2}}\sum_{x_{t}}\sum_{x_{t}}k_{\sigma }\left(x,x_{i}\right)=\frac{1}{N^{2}}I^{T}KI,$$

where I denotes the (N×1) vector and K denotes the (N×N) kernel matrix. Now, the quadratic Renyi entropy can be approximated using the sample’s kernel matrix. In the diagonalization of the kernel matrix *K*, $K=EDE^{T}$, in which $D=diag\left(\lambda_{1},\ldots ,\lambda_{N}\right)$ is a diagonal matrix storing the eigenvalues and *E* is a matrix with the eigenvectors [23,24]. Substituting $E=\left\{e_{1},\ldots ,e_{N}\right\}$. into Equation (3), we have:

(4) $$\hat{V}\left(p\right)=\frac{1}{N^{2}}\sum_{i=1}^{N}\left(\sqrt{\lambda_{i}}e_{i}^{T}1\right),$$

From Equation (4), it can be seen that the contribution to Renyi entropy varies with the eigenvalues and eigenvectors. Therefore, during the kernel entropy analysis, by selecting the top one eigenvalue and feature vectors with most contributions to the Renyi entropy, the data of the feature space, i.e., the principal component matrix $\varphi_{eca}=D_{i}^{\frac{1}{2}}E_{i}^{T}$, can be obtained. Afterwards, we calculate the inner product of the data points in the feature space as $K_{eca}=\varphi_{eca}^{T}\phi_{eca}$.

### 2.2. Definition of the KECA-based Similarity Metric

For nonlinear batches, phase localization should be checked by revealing the changes in nonlinear behaviors throughout the operation duration. To better capture the multiplicity of nonlinear phases, the clustering should be implemented in the mapped high dimensional feature space, where the nonlinearity underlying process measurement has been converted into linearity. Then, the key is to determine which analysis object can be used to reveal the changes of inherent nonlinear behaviors. Consider the three-way reference data $X\in R^{N}$ By mapping the data into the feature space *F* via KECA, we have:

(5) $$\left\{\varphi :R^{N}\to F,x\to \varphi \left(x\right)\right\},$$

where $\phi =\left\{\phi_{\left(x_{1}\right)},\ldots ,\phi_{\left(x_{M}\right)}\right\}$. By defining Equation (4) as the criterion for the choice of KECA mapping direction, the projection vector P is given as follows:

(6) $$P=\frac{1}{\sqrt{\lambda_{i}}}\varphi e_{i},$$

The similarity is defined in Equation (7) to measure the degree of similarity between two projection vectors:

(7) $$D=diss\left(P_{1},P_{2}\right)=\frac{4}{J}{\sum_{j=1}^{J}\left(\lambda_{1}^{j}-0.5\right)}^{2}=\frac{4}{J}\sum_{j=1}^{J}{\left(\lambda_{2}^{j}-0.5\right)}^{2},$$

It can be seen that *D* ranges from 0 to 1. Given the kernel entropy load matrices $P_{1}$ and $P_{2}$, if the value $\lambda_{1}^{j}$ approaches 0.5, it indicates a high level of similarity between them; if it approaches 1 or 0, it means a high level of dissimilarity.

The KECA-based similarity measures the similarity between two kernel entropy load matrices. The smaller the value of *D* is, the higher the level of similarity between the two matrices becomes. The standardized data is extended along the direction of the variable and then it is mapped on the high-dimensional space through KECA mapping, by constructing a projection vector *P*. Similarity $P_{k}\left(I\times J\right)$ of the two neighboring kernel entropy load matrices is computed. The value of the similarity metric is used as the input of the clustering algorithm. By clustering the data using the load matrices of all batches, the proposed method fully utilizes the non-linear data. The process duration is thus properly divided into different nonlinear phases, making it very robust by effectively overcoming the disruption from a few batches.

### 2.3. Phase Partitioning Based on the KECA Similarity

**Figure 2:**
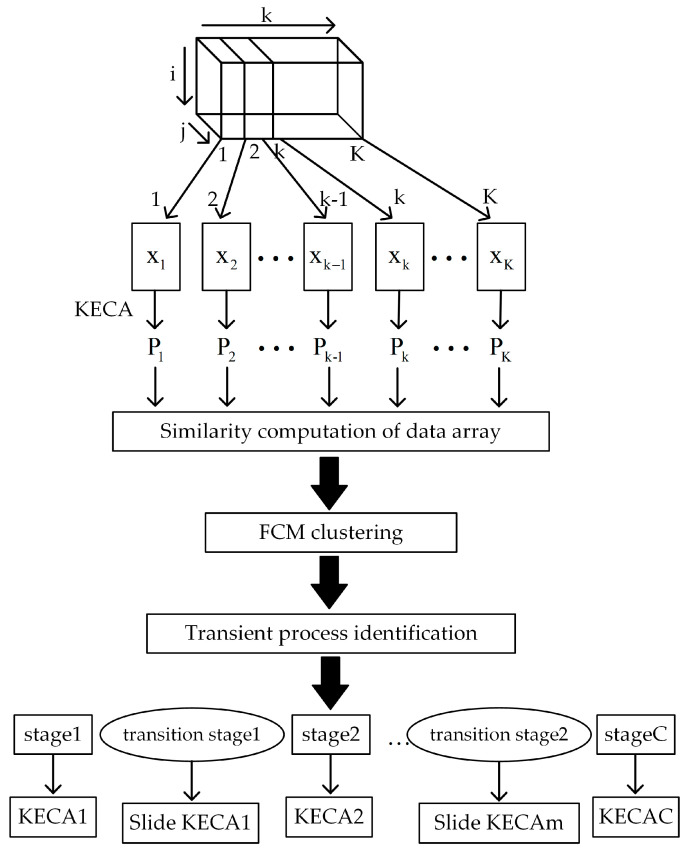
Model of the partitioned phases [25].

Based on the KECA similarity, the sub-phases are partitioned into stable ones and transitional ones. Because the time slices during all sub-phases have the same characteristics, the data in the same time period can be described using the same model. Figure 2 illustrates the principles of the phase partitioning algorithm, whose steps are detailed below:

(1) Extend the matrix of the three-way model data in the direction of the batch and standardize the matrix. Perform vertical cutting in the direction of time to produce and standardize the time slice matrix.
(2) Map each of the time slice matrices to the high-dimensional feature space using the kernel entropy. Let $P_{i}$ denote the load matrix, representing the correlation between procedure variables.
(3) For each $P_{i}=\left(I\times J\right),i=1,2,\ldots ,K$, compute the similarity $D_{i}=\left(k\right)=diss\left(P_{i},P_{j}\right)$, where $\{\begin{array}{c} k=j,j=1,2,\ldots ,i-1\\ k=j-1,j=i+1,\ldots ,K \end{array}$, and $D_{i}$ is the input sample of the cluster.
(4) Perform the preliminarily partitioning of the phases using the fuzzy C-mean clustering (FCM) algorithm. Firstly, partition the process into c phases according to the rule of maximum membership degree. Afterwards, detect the outliers having the maximum membership degree during each phase, using the single-variable control diagram. The succession of outliers mostly occurs at the start or end of a phase. This can be used as the criterion to determine the start and the end of the transitional phase. Finally, after the transitional phase is eliminated, the remaining phase is the stable phase.
(5) Determine the control limit of the single-variable control diagram through several iterations, i.e., obtain the control limit for the current data in iteration using the traditional single-variable control diagram. Then, remove the outliers beyond the control limit and update the data. Repeat these steps until the control limit converges. Since the control limit is determined using the local statistical method rather than subjectively, the transitional phase is determined in a more objective and reasonable manner.

## 3. Phase-Wise Monitoring and Diagnosis Based on KECA Similarity

### 3.1. CS Statistic

KECA was originally designed for spectral classification. The samples having similar features under certain similarity metrics are classified into the same type and dissimilar samples are classified into other types. Different types of data have different angles with the origin of the kernel feature space after KECA transformation. If the utility function of a cluster is able to determine such an angular structure, it can be very beneficial for distinguishing between normal and abnormal data. Therefore, an adequate fault monitoring statistic is an absolute necessity. The divergence statistic, also known as Cauchy-Schwarz (CS) statistic, is able to measure the distance or similarity between two probability density functions *p*1(*x*) and *p*2(*x*) [26,27]. Transforming it into the kernel space yields the cosine of the angle between vectors. Due to its remarkable ability to represent the angular distance of data features extracted through KECA, it can effectively distinguish between normal and abnormal data. For the data of the three-way batch process, the CS statistic is computed as:

(8) $$CS=1-\cos∠\left(M_{k},M_{k}^{i}\right)=1-\sum_{j=1}^{l}\frac{m_{k,j}^{T}m_{k,j}^{i}}{\left\|m_{k,j}\right\|\left\|m_{k,j}^{i}\right\|},$$

where $m_{k}^{i}=\left[m_{k,1}^{i},m_{k,2}^{i},\ldots ,m_{k,j}^{i}\right]$ denotes the principal component matrix of the *i*-th batch of data at the *k*-th sampling moment, $M_{k}=\frac{1}{I\sum_{i=1}^{I}M_{k}^{i}}$ denotes the mean of the principal component matrix of the *i*-th batch at the *k*-th sampling moment and $M_{k}=\left[m_{k,1},m_{k,2},\ldots ,m_{k,l}\right]$ denotes the number of principal components.

Under the normal operation, the online and the old models are very similar and thus produce a small statistic [28]. Once the error happens, the two models differ considerably and the degree of similarity decreases quickly. On the other hand, the CS statistic decreases rapidly to a level higher than the control limit, and the fault is detected then. The CS statistic’s control limit R is computed using the kernel density approximation method [29].

### 3.2. Model of the Stable Phase

After the partitioning is performed in the stable and transitional phases, a KECA model is established for each of the stable phases. Steps are given below:

(1) Extend the three-way reference data matrix $X\left(I\times J\times K_{c}\right)$ in the direction of the batch and then vertically cut the matrix in the time direction to yield the time slice matrices. Normalize each of the time slice matrices. Combine the normalized data of all time slices during each stable phase together and extend it into a 2-D matrix $X_{c}\left(Ik_{c}\times J\right)$, where $k_{c}$ denotes the number of sampling points collected in the *c*-th stable phase. Normalize the data.
(2) Establish the KECA model for $X_{c}\left(Ik_{c}\times J\right)$ to obtain the principal component matrix. Set the kernel function and kernel parameters. Compute the kernel matrix *K* for each of the pre-processed time slice matrices. Obtain eigenvalues and feature vectors of the kernel matrix through matrix decomposition. Obtain the Rayleigh entropy corresponding to each eigenvalue based on Equation (4). According to the Rayleigh entropy procedure, choose the top *l* principal component vectors to constitute the principal component matrix.
(3) Determine the control limit of the statistic. Compute the statistic CS based on Equation (8) at each moment k. Obtain the control limit R1 of CS through the kernel density approximation.

### 3.3. Modelling of the Transitional Phase

(1) Arrange the three-way reference data $X_{m}\left(I\times J\times k_{m}\right)$ of each transitional phase into the 2D data matrix $X_{m}\left(Ik_{m}\times J\right)$ in the direction of the variable, where $k_{m}$ denotes the number of sampling points at the transitional phase *m*.
(2) Establish a sliding KECA model using the input samples $X_{m}$. In general, at the early stage of the transition, the process at each time instance is similar to the previous phases. Afterwards, at the late stage of the transition, the process changes to contain the status of the next phase. Hence, we can establish a weighted KECA model for $X_{m}\left(Ik_{m}\times J\right)$ at the transitional phase, yielding a principal component matrix M, and $M=\lambda M_{1}+\left(1-\lambda \right)M_{2}$, where $M_{1}$ denotes the principal component matrix obtained from the KECA model constructed at the stable phase before the transitional phase starts, $M_{2}$ denotes the principal component matrix obtained from the model constructed in the subsequent stable phase after the transitional phase ends. $\lambda =\frac{X^{\prime }}{X_{2}-X_{1}}$, where $X^{\prime }$ denotes the time instance at the current transitional phase, $X_{1}$ and $X_{2}$ denote the ending time of the previous stable phase and the starting time of the subsequent stable phase. This enables the model to make transition from the previous stable phase to the next stable phase.
(3) Determine the control limit of the statistic. Compute the statistic CS at each time moment *k* based on Equation (8). Obtain the control limit R2 of CS through kernel density approximation.

### 3.4. SV-KCP-based Fault Diagnosis

Due to the incapability of finding a reverse mapping from the high-dimensional feature space to the low-dimensional input space, it is impossible to derive a formula for computing the contribution of the statistic. Therefore, the traditional contribution plot is not appropriate for the kernel space mapping method.

To address the problem stated above, a standard vector-based kernel space contribution plot method (SV-KCP) is proposed in this paper. Like the traditional contribution plot, the proposed method executes effortlessly, intuitively and without any fault samples. More importantly, the proposed method eliminates the need to derive the exact formula, making it theoretically appropriate for kernel mapping approaches, such as KPCA and KICA.

**Figure 3:**
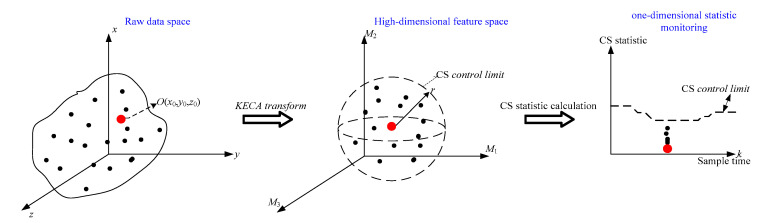

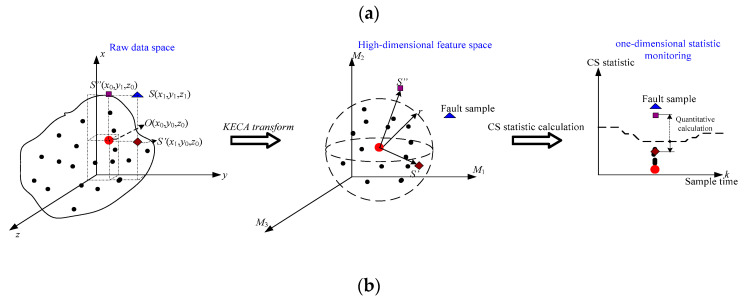
Principles of SV-KCP. (**a**) Normal operating condition; (**b**) Abnormal condition [25].

Figure 3 shows the principles of SV-KCP. Consider a data space with three initial dimensions which is mapped to the high-dimensional feature space using KECA, with three principal components retained. As seen in Figure 3a, the normal data set is mapped to the feature space at any time moment *k* during the batch process. Assume that the mapping data is clustered within a sphere of radius *r*. Further calculation of the statistic CS indicates that the new data is completely under the CS control limit.

Now, suppose that there is a central vector in the feature space, which is located at the mass center of this sphere. Its corresponding point in the original data space is denoted by the point *O* in Figure 3. The vector from *O* is considered to be the standard vector. If a fault is detected at time moment *k*, each of the variables belonging to the fault’s original samples is iteratively used as a substitute for the corresponding variable of the standard vector *O* at time moment *k*. Afterwards, the process status is again monitored using the new input samples, i.e., KECA projection—CS statistic calculation of difference with the control limit. In this way, the contribution of each variable to the statistic can be described quantitatively. From the control limit, it can be determined whether the contribution is within a reasonable range, providing more remarkable insights into the detection of the reason behind the fault. From Figure 3b, it is intuitive to see whether the statistic exceeds the limit after the variables x and y of *O* are replaced respectively.

The problem is now transformed to determining the standard vector of the point *O*. Clearly, due to the impossibility to obtain the reverse mapping from feature space to original space, it is impossible to get the vector of original data from the mass center vector of the feature space. However, from Equation (8), it can be seen that among the $M_{k}^{i}$. samples, the one with the smallest value of CS is the closest one to the feature space’s mass center vector. Hence, the data in the original space corresponding to this sample can be used as an approximation of the standard vector of *O* at the moment *k*.

## 4. Simulation Experiment

### PenSim Simulation Platform

The process monitoring and technology team from the Illinois Institute of Technology developed the penicillin manufacture simulation platform PenSim2.0 [30]. It provides a baseline application for process monitoring and fault diagnosis of the penicillin’s batch process. The reaction time of the fermentation process is 400 h for each batch of penicillin, and the sampling interval is 1 h. The process is monitored using 10 variables, including the ventilation, agitation power, substrate feed rate, oxygen concentration in the water, fermenter capacity, carbon dioxide concentration, pH value, fermenter temperature, culture volume, and cold flow velocity as shown in Table 1. In order to further match the actuality, all measuring variables are degraded with measurement noises. A total of 35 normal batches are generated as the reference database of the original model, producing the three-way reference data matrix *X*(35 × 10 × 400). The matrix is then pre-processed in the direction of the batch and partitioned into 400 time slice matrices *Xi*(35 × 10). The similarity *Di* is then computed from Equation (7) as the input sample of the cluster.

**Figure 4:**
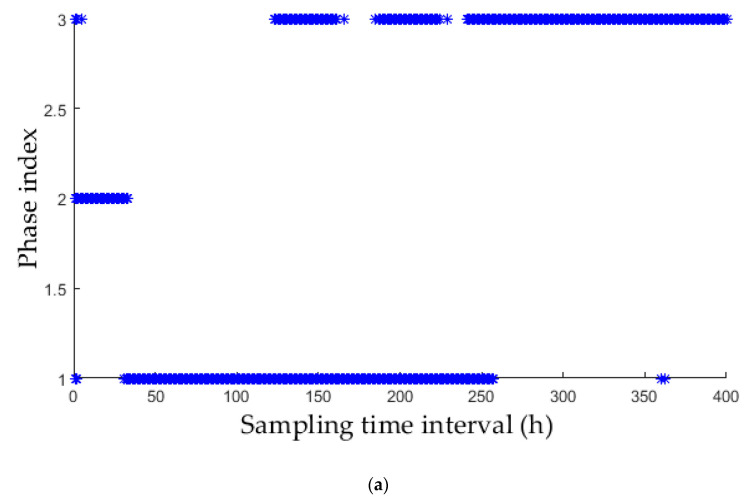

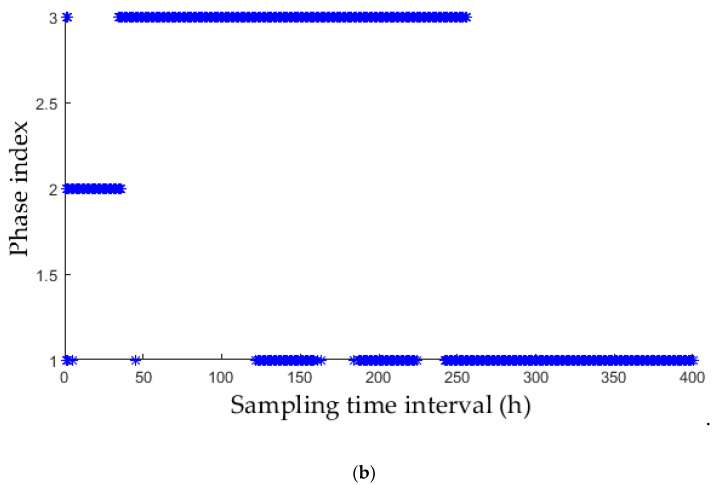
Phase division results using original data. (**a**) K-means clustering result; (**b**) FCM clustering result.

In order to show the validity of this chapter based on KECA similarity, compare the segmentation results of traditional clustering algorithm. As shown in Figure 4, the data vector of each batch is taken as input sample. Figure 4a uses the *k*-means algorithm to cluster the data. Figure 4b original data is a direct clustering algorithm using FCM algorithm. From the two charts, it is clear that the results of classification are not ideal, and there is almost no way to classify the second and third stages. Figure 4a shows the result of preliminary clustering using the KECA-based similarity metric *Di*. Obviously, the classification result is desirable and well matches the mechanism of fermentation. This indicates that during penicillin fermentation, the correlation between procedure variables does not fluctuate with time. Instead, it varies with the characteristics of the process’ mechanism at different phases.

**Figure 5:**
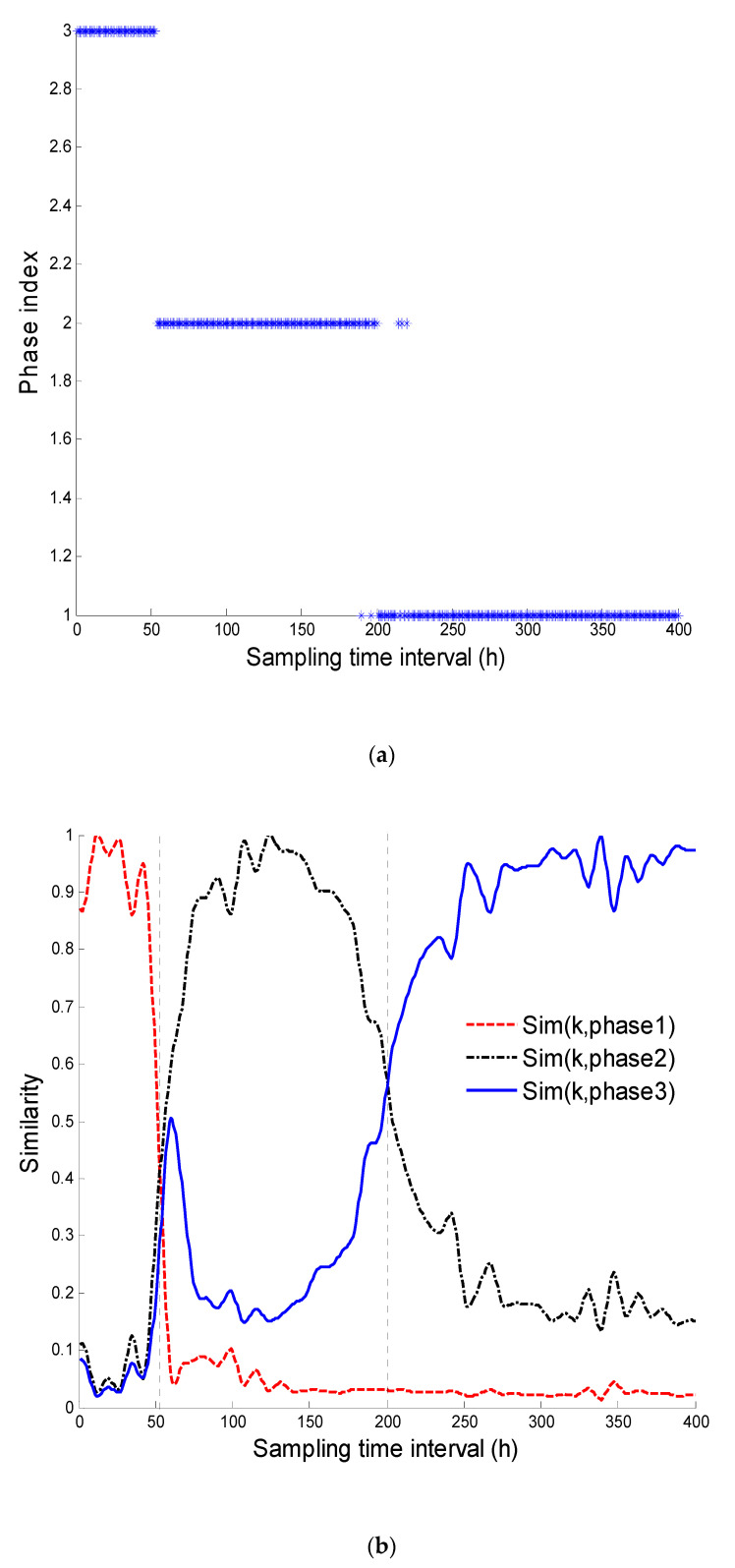

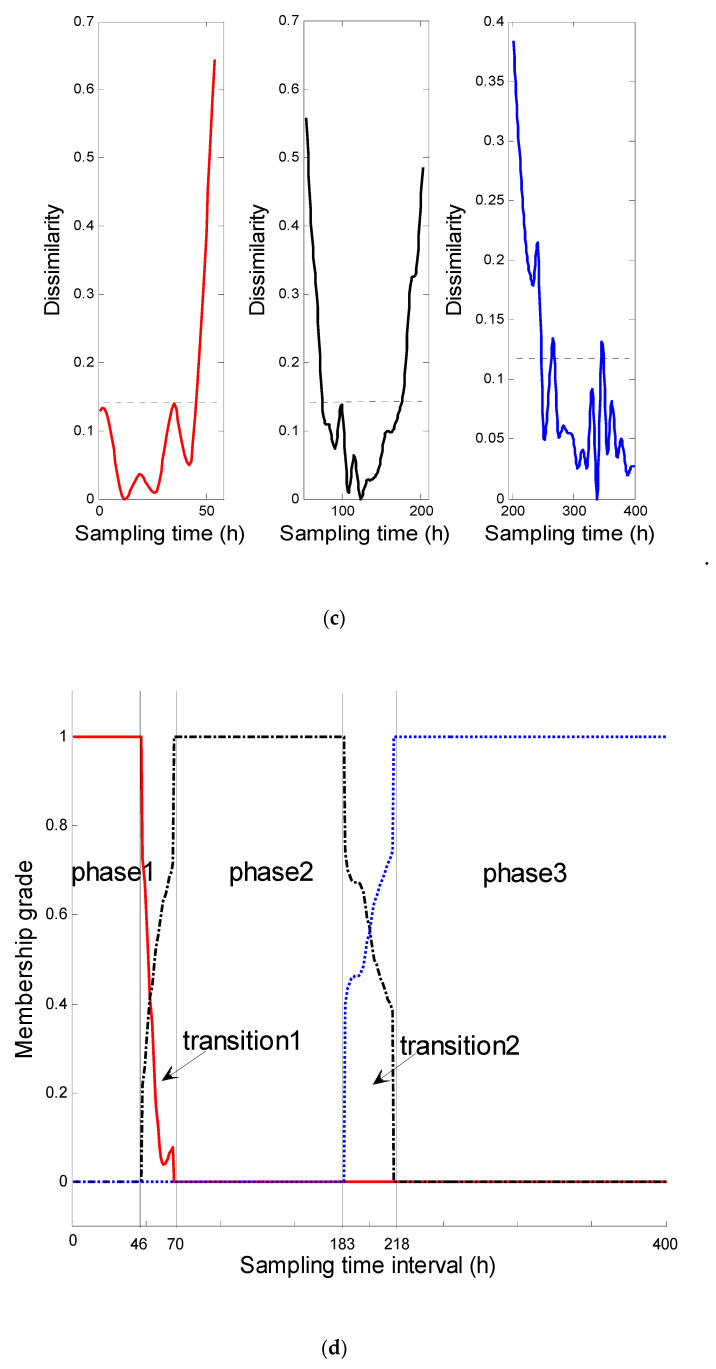
Phase division result using the proposed method. (**a**) FCM clustering result; (**b**) Membership grades; (**c**) Transition ranges identification; (**d**) Sketch map of the similarity [25].

In Figure 5b, Sim(*k*,*c*) denotes the membership degree of the *k*-th time slice matrix with respect to the *c*-th phase. The figure shows the changes with the process’ operation or characteristics of the mechanism. In the middle stage of the phase c, Sim(*k*,*c*) (k∈c) is approximated to 1, revealing a high level of similarity between the current data block and that stage. In the start and the end of phase c, it decreases gradually, indicating the existence of transition between neighboring phases. After the preliminary division of sub-phases, the transition phase is identified using the single-variable control diagram, as shown in Figure 5c. The dissimilarity of the data block, 1-Sim(k,c), is computed for each phase and then taken as an input in the single-variable control diagram. The range of the transition process is determined by detecting the sequence of outliers in the start and the end of the phase.

Figure 5d intuitively shows the characteristics of transition between successive phases. Compared with the traditional clustering algorithm, process duration is properly divided into different nonlinear phases, making it very robust by effectively overcoming the disruption from a few batches. The sampling interval obtained in the way above is (1~48), (70~188) and (215~400) for the stable phase, (49~69) and (189~214) for the transitional phase. The monitoring model of each phase is then established.

In order to demonstrate the effectiveness of the proposed algorithm, multiple fault batches are generated covering a variety of procedure variables and fault types, as shown in Table 2. Each type of fault corresponds to three fault batches with different initial values and amplitudes. The mean value is defined as the final evaluation metric of this fault type. Comparison is made with MPCA and sub-PCA [6].

**Figure 6:**
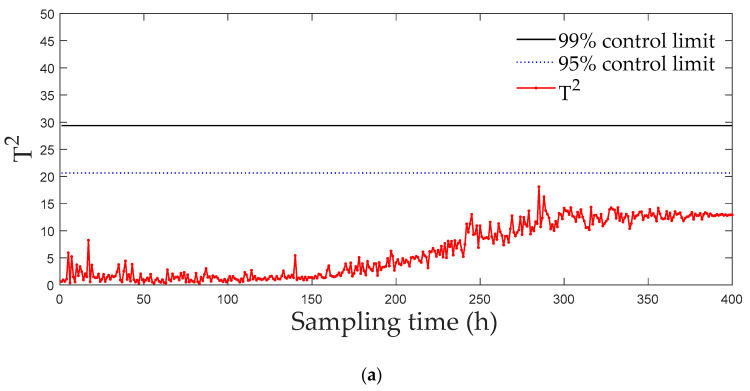

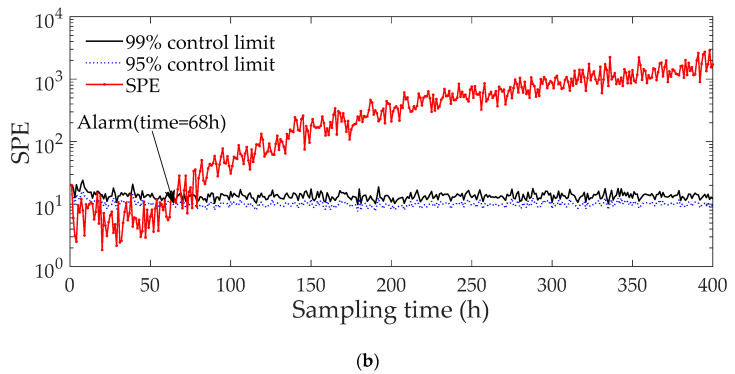
Monitoring results using MPCA for fault 5. (**a**) The monitoring results of the statistics T2; (**b**) The monitoring results of the statistics SPE [25].

**Figure 7:**
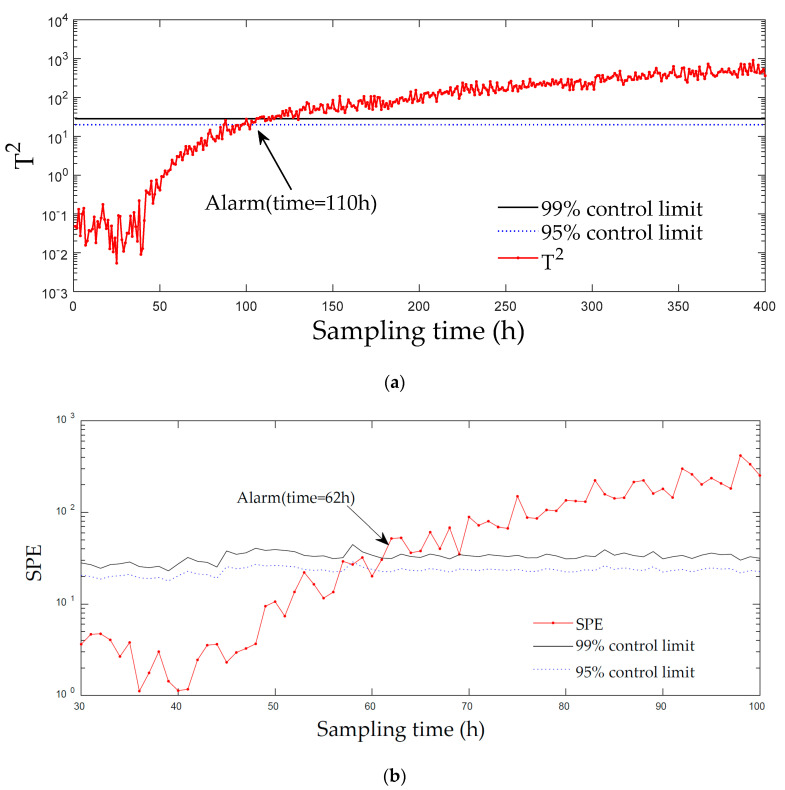
Monitoring results using sub-PCA for fault 5. (**a**) The monitoring results of the statistics T2; (**b**) The monitoring results of the statistics SPE [25].

**Figure 8:**
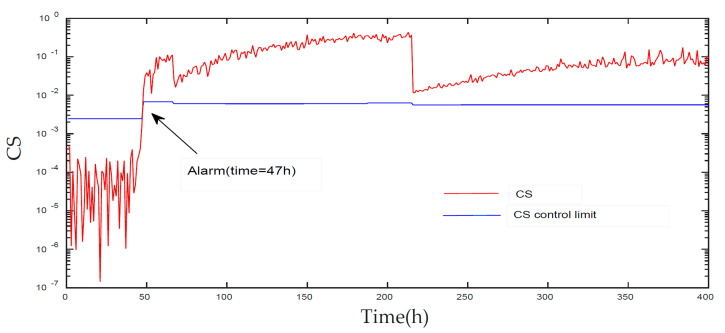
Monitoring results of fault 5 using KECA after phase partitioning [25].

**Figure 9:**
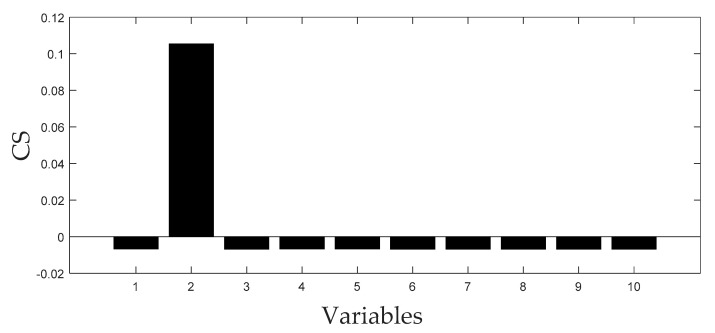
CS of fault 5 using SV-KCP.

Figure 6 and Figure 7 displays the monitoring results of the statistics T2 and SPE on the fault type 5, using MPCA and sub-PCA, respectively. Figure 8 shows the statistic CS monitored using the proposed method. In this type of fault, slope disturbance with a gradient of 1.2% is added to the agitation power at the 47th h and then lasts until the reaction ends. From the figure, it can be seen that the proposed method detected the anomaly in the 47th h, almost as soon as the fault happened, about 21 h and 15 h earlier than MPCA and sub-PCA, respectively. In the case of T2 monitoring, sub-PCA detected the fault about 55 h later than the proposed method and MPCA failed to detect any anomaly. Analysis indicated that this fault happens during the transition phase 1. The sub-PCA method strictly partitions the phase into different sub-phases and ignores the non-linear of process, thereat it is unable to effectively reflect the process characteristics. Compared with the nonlinear phase fault monitoring in this paper, it has a long delay in fault detection. Regarding the data of the entire batch as a whole, MPCA cannot accurately represent the characteristics of all phases or alternatively cover the operation range of all phases. The control limit is thus too unclear, making it unable to generate warning in the case of malfunction during certain phases, resulting in many alarm omissions. Therefore, variation of the characteristics in the transition phase has a lot of influence on the monitoring result and it must be taken into account. Fault detection is followed with fault identification and diagnosis. Figure 9 shows the contribution plot obtained from SV-KCP. It can be seen clearly from the figures that the fault is attributed to anomaly of the second variable, agitation power.

**Figure 10:**
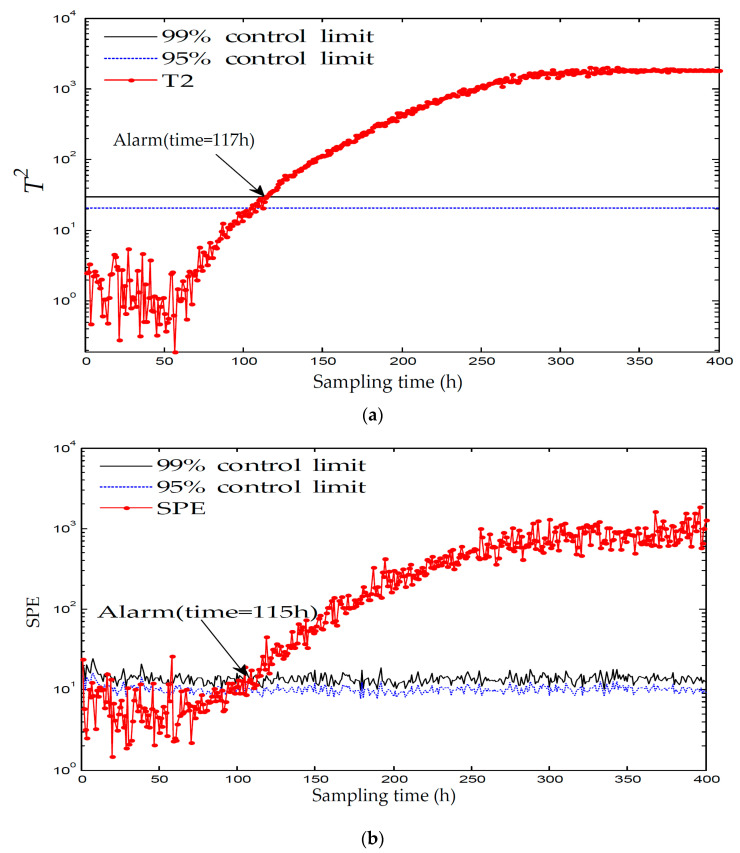
Monitoring results using MPCA for fault 1. (**a**) The monitoring results of the statistics T2; (**b**) The monitoring results of the statistics SPE

**Figure 11:**
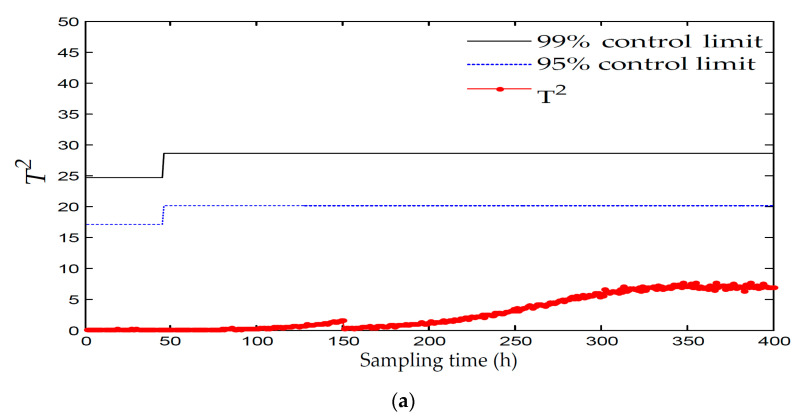

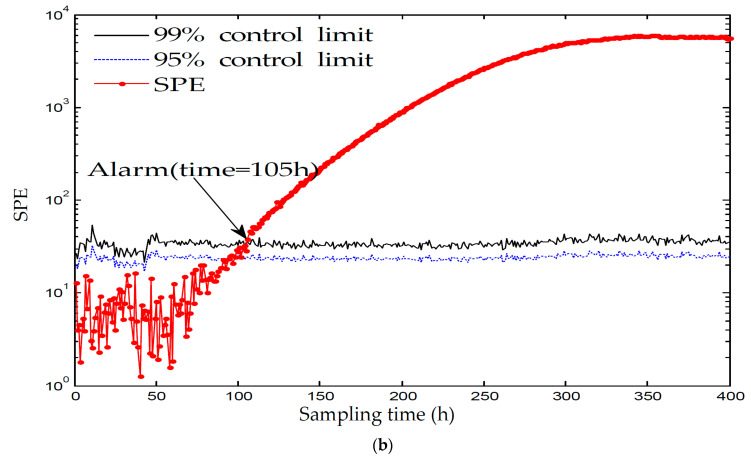
Monitoring results using sub-PCA for fault 1. (**a**) The monitoring results of the statistics T2; (**b**) The monitoring results of the statistics SPE.

**Figure 12:**
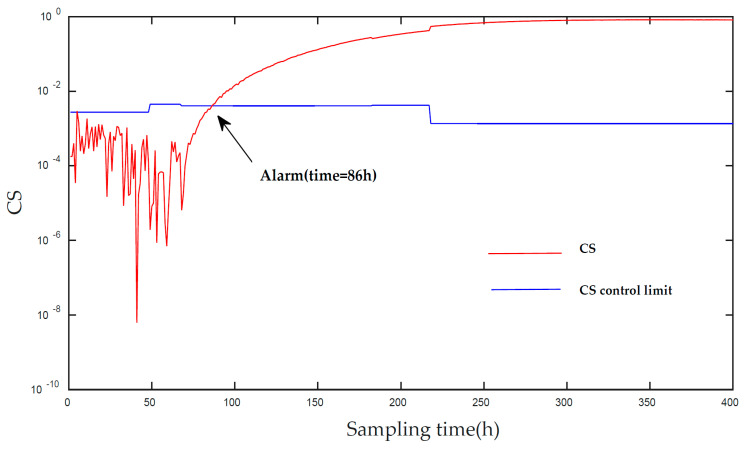
Monitoring results using the proposed method.

**Figure 13:**
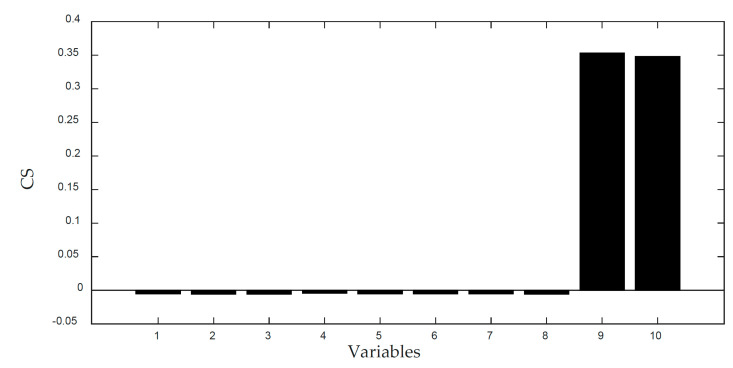
CS of fault 1 using SV-KCP.

Figure 10 and Figure 11 displays the monitoring results of the statistics T2 and SPE on the fault type 1, using MPCA and sub-PCA, respectively. Figure 12 shows the statistic CS monitored using the proposed method. In this type of fault, the step disturbance which reduces the supply rate by 15% is added to the substrate supply rate at the 70th h and then lasts until the reaction ends. From the figure, it can be seen that the proposed method detected the anomaly in the 86th h, about 29 h and 19 h earlier than MPCA and sub-PCA, respectively. In the case of T2 monitoring, sub-PCA failed to detect any anomaly. In addition, in the beginning of fermentation, the MPCA method showed more false alarm phenomena. It can be seen that the traditional linear clustering algorithm is not ideal for nonlinear multi-stage intermittent process. In general, the indirect effect of substrate supply rate changes the normal process of fermentation. It is mainly reflected by the changes in the parameters such as the volume of the culture volume and the cooling water flow rate. Figure 13 shows the contribution plot obtained from SV-KCP. It can be seen clearly from the figures that the fault is attributed to anomaly of nine and ten variable, culture volume and cooling water flow rate. Thus, the fault is caused by substrate feed rate.

Table 3 compares the monitoring performance of three methods. It can be observed that the proposed method detects all faults effectively, providing the lowest false alarm rate. Hence, the proposed method is able to monitor the process more robustly.

## 5. Industrial Applications

**Figure 14:**
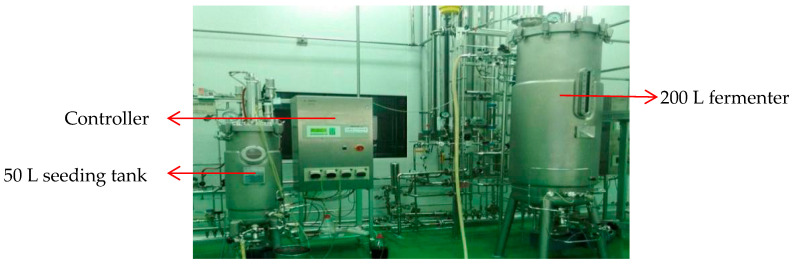
Illustration of the *E. coli* fermentation system.

In the pharmaceutical industry, continuous fermentation of transgenic *E. coli* in batches is widely used for the production of pharmaceutical proteins. *E. coli* fermentation is a series of very complex biochemical reactions, characterized by multi-variable coupling, strong non-linearity, time dependence and uncertainty. In this section, the proposed method is applied to monitor the process of fermentation in a Beijing Yizhuang Pharmaceutical Corporation, focusing on the production of interleukin–2 from transgenic *E. coli* exogenous protein expression. Figure 14 shows the 50 L fermenter. The system uses the peristaltic pump to control the aeration rate, mixing speed, temperature and medium supplying rate (dextrose and ammonia). The entire fermentation period lasts about 19–20 h. The first phase is approximately 5–6 h long for bacteria adaptation after the table nurtured inoculation is performed. The second phase lasts about 3.5 h, during which the sugar concentration needs to be maintained at a high level in the fermenter due to high glucose consumption. The last phase is 8–9 h long, during which the sugar concentration needs to be maintained at a moderate level to facilitate exogenous protein expression.

**Figure 15:**
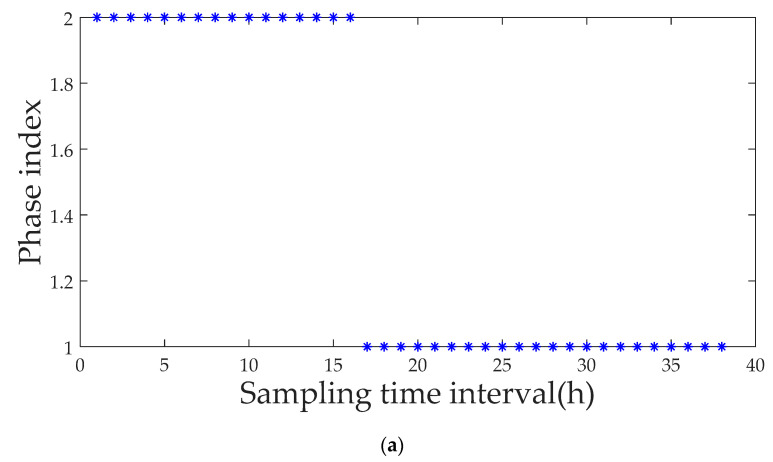

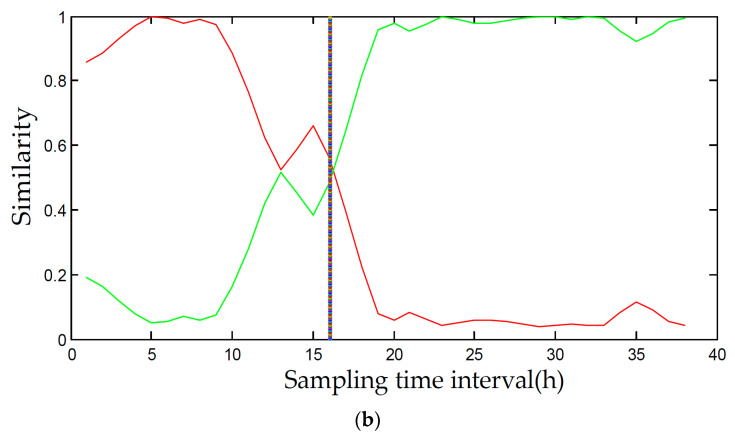
Phase division result using the proposed method. (**a**) Phase division result using phase index; (**b**) Phase division result using similarity index.

The experiment lasts 19 h with a sampling interval of half an hour. The nine major procedure variables chosen to measure the bacteria growth and exogenous protein expression include pH value, dissolved oxygen concentration, fermenter pressure, temperature, mixing speed, glucose supply, culture medium supply, and ventilation. A total of 33 normal batches are selected as the model’s reference database, yielding a three-way reference data matrix *X*(33 × 9 × 38). The phases are then partitioned using the proposed method. All of the phases and the sampling interval of the transition process finally determined are shown in Figure 15, (1–10) and (18–38) for the stable phase and (11–17) for the transitional phase.

**Figure 16:**
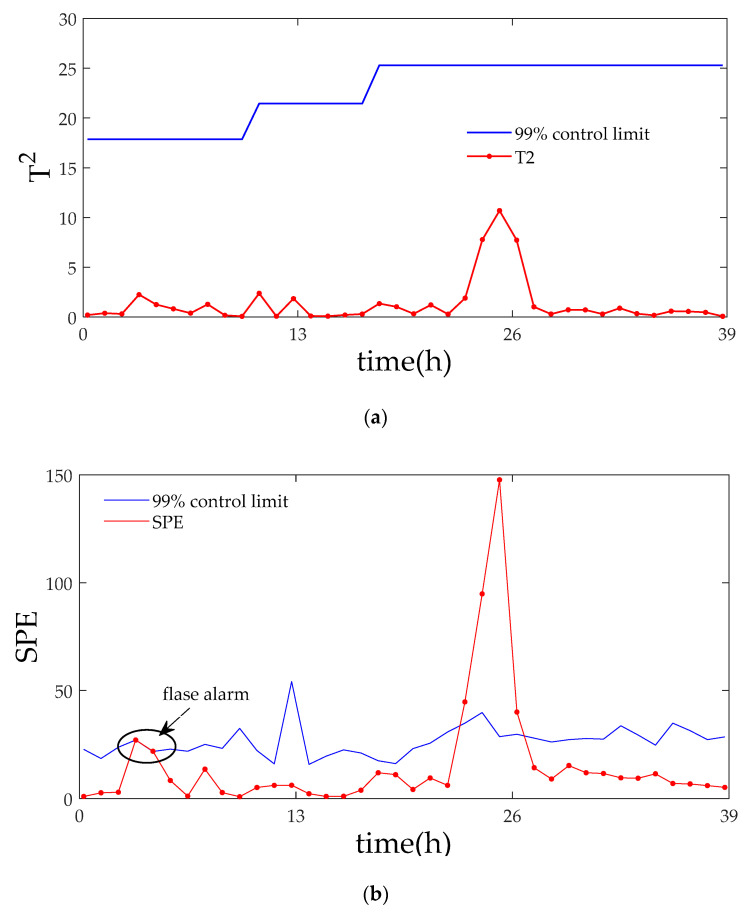
Monitoring results using sub-PCA for fault 2. (**a**) The monitoring results of the statistics T2; (**b**) The monitoring results of the statistics SPE.

**Figure 17:**
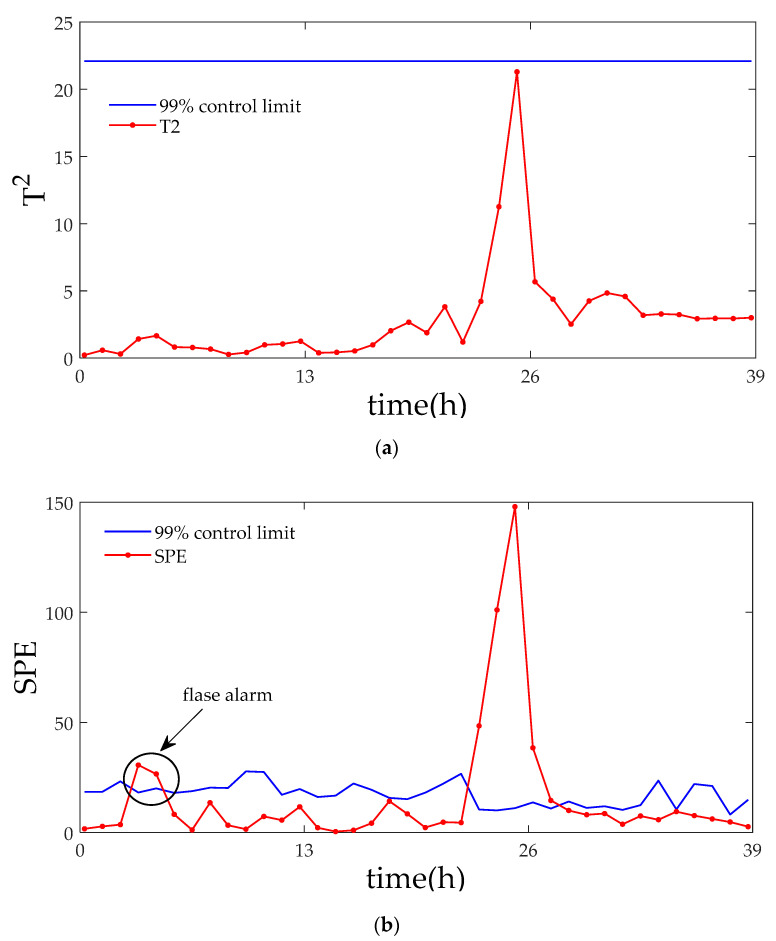
Monitoring results using MPCA for fault 2. (**a**) The monitoring results of the statistics T2; (**b**) The monitoring results of the statistics SPE.

**Figure 18:**
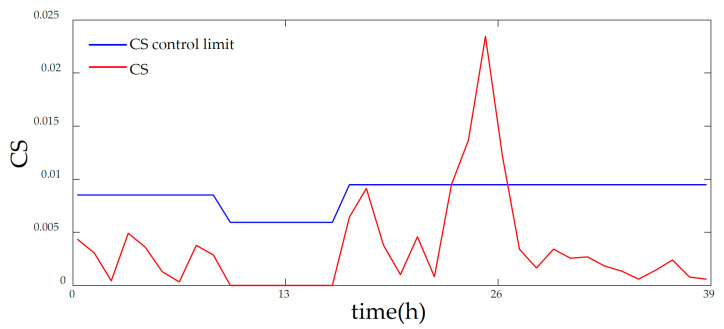
Monitoring results using the proposed method after phase partitioning.

**Figure 19:**
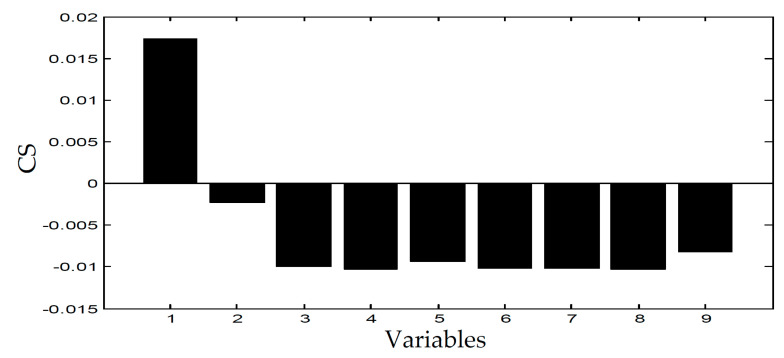
Diagnosis results using SV-KCP for fault 2.

Figure 16, Figure 17 and Figure 18 contain the monitoring results of the production process with the second type of fault using sub-PCA, MPCA and the proposed method, respectively. It can be seen from SPE monitoring that both sub-PCA and MPCA detect the fault at the 23rd h, but they make false alarms in varying degrees. In terms of T2 monitoring, neither MPCA nor sub-PCA detected any anomaly. On the contrary, the proposed method quickly detected the fault at the 11th h and created no false alarms, thereby revealing the product’s quality accurately. Figure 19 shows the monitoring result of the proposed method at the stable phase 3. Diagnosis conclusion is reached from the contribution plotting that the fault should be attributed to the anomaly of pH value.

Through the application of the above fermentation process, it can be seen that this method in this paper can realize the fault monitoring of batch fermentation process, reduce the false alarm rate and false failure alarm rate of the process, improve the production efficiency of the process and accurately detect the variables causing the failure.

## 6. Conclusions

A multistage fault monitoring and diagnosis strategy, based on KECA similarity, is presented for batch processes with heavy nonlinearities. The nonlinear features of processes are extracted effectively with the proposed method, based on which the division of steady phases and transitions are achieved. In terms of the phase division results, different KECA models and slide-KECA models are established, respectively. SV-KCD is simultaneously proposed in this paper for fault diagnosis to ensure the operation safety. In the penicillin experiment, our strategy shows more satisfactory performance, not only in shortening the detection time but also in avoiding the occurrence of missing alarms and false alarms, in comparison with the traditional methods. Furthermore, with SV-KCD in hand, our method can diagnose the main fault causing variables precisely and in time. When applied to real industrial processes, the proposed method can better reflect the diversity of features in different stages, which shows the certain practical value of our method for solving the problem of fault monitoring in multistage batch process. In the future, more effective algorithms should be integrated into the development of process monitoring application software and realize the application in the actual industrial process.

## Figures and Tables

**Table 1:** Variables used in the monitoring of the Penicillin fermentation process.

Number	Process Variable	Number	Process Variable
1	aeration rate (L·h^-1^)	6	carbon dioxide concentration (g·L^−1^)
2	agitator power (W)	7	pH
3	substrate feed rate (K)	8	fermentor temperature (K)
4	dissolved oxygen concentration (%)	9	culture volume (L)
5	substrate feed temperature (kcal)	10	cooling water flow rate (L·h^−1^)

**Table 2:** Fault types in the simulation.

Fault Numbers	Procedure Variables	Fault Types
1	Substrate supply rate	Step disturbance
2	Agitation power	Step disturbance
3	Ventilation rate	Step disturbance
4	Substrate supply rate	Slope disturbance
5	Agitation power	Slope disturbance
6	Ventilation rate	Slope disturbance

**Table 3:** Comparison of monitoring results from three results [25].

Working Condition	Error Rate of Type I (%)			Error Rate of Type Ⅱ (%)		
	MPCA	Sub-PCA	KECA	MPCA	Sub-PCA	KECA
Normal	5.71	2.51	1.78	—	—	—
Fault 1	1.54	0.79	0.67	13.46	43.08	6.8
Fault 2	5.69	1.43	0.47	6.97	0	0
Fault 3	7.72	2.8	1.27	12.33	0.9	0.78
Fault 4	3.84	1.89	1	23.4	41.7	5.2
Fault 5	3.13	1.36	1	8.9	38.9	2
Fault 6	3.22	0.76	0.75	55.25	42.5	3.2
